# Coverage, Formulary Restrictions, and Affordability of Sodium-Glucose Cotransporter 2 Inhibitors by US Insurance Plan Types

**DOI:** 10.1001/jamahealthforum.2021.4205

**Published:** 2021-12-17

**Authors:** Sri Lekha Tummalapalli, Julio Lamprea Montealegre, Neil Warnock, Michael Green, Said A. Ibrahim, Michelle M. Estrella

**Affiliations:** 1 Division of Healthcare Delivery Science and Innovation, Department of Population Health Sciences, Weill Cornell Medicine, New York, New York; 2 Division of Cardiology and Kidney Health Research Collaborative, University of California, San Francisco; 3 Medical Affairs - Market Access, Bayer AG, Whippany, New Jersey; 4 Fingertip Formulary, Clarivate Analytics (US) LLC, Chandler, Arizona; 5 Kidney Health Research Collaborative, Department of Medicine, San Francisco Veterans Affairs Health Care System and University of California, San Francisco, San Francisco

## Abstract

This cross-sectional study evaluates the stipulations of coverage under commercial, health insurance exchange, Veterans Affairs, Medicare, Medicaid, or other insurance for canagliflozin, dapagliflozin, empagliflozin, and ertugliflozin.

## Introduction

Sodium-glucose cotransporter 2 (SGLT2) inhibitors reduce the risk of kidney failure, cardiovascular events, and death in patients with chronic kidney disease and/or heart failure, regardless of diabetes status. Despite high-quality evidence and guideline recommendations supporting their use, uptake of these medications remains low.^1^ Limited insurance coverage, formulary restrictions, and high out-of-pocket costs may represent barriers to SGLT2 inhibitor use. Coverage of SGLT2 inhibitors has been described in Medicare Part D plans but not in commercial, employer-based, or Medicaid plans.^2^ We examined the insurance coverage, formulary restrictions, and cost sharing for SGLT2 inhibitors across plan types in the US.

## Methods

We followed the Strengthening the Reporting of Observational Studies in Epidemiology (STROBE) reporting guideline. This study did not involve human participants and therefore did not require institutional review board review or informed consent, according to University of California, San Francisco institutional policy.

Plan-level coverage information for the SGLT2 inhibitors dapagliflozin, canagliflozin, empagliflozin, and ertugliflozin was obtained from Fingertip Formulary (Clarivate Analytics LLC), reflecting coverage in April 2021. Fingertip Formulary maintains detailed drug coverage information for more than 5000 health plan and employer formularies in all 50 states, the District of Columbia, and Puerto Rico.^3,4^ Plan type was classified as (1) commercial or employer-based; (2) health insurance exchange (HIX), Veterans Affairs (VA), or other (including Federal Employees Health Benefits program, non-VA federal program, municipal plan, pharmacy benefit manager, and union); (3) Medicare; and (4) Medicaid. The study outcomes were insurance coverage (yes or no); formulary restrictions, including prior authorization (yes or no), step therapy (yes or no), and quantity limits (yes or no); and cost sharing, including co-payments and co-insurance (%). A summary variable was constructed for plan coverage of at least 1 SGLT2 inhibitor without prior authorization or step therapy.

For each SGLT2 inhibitor, we used χ^2^ tests to compare the differences in coverage and formulary restrictions and used unadjusted linear regression to compare cost sharing by plan type. All analyses were weighted by covered pharmacy lives. Analyses were performed using Stata/IC, version 15.1 (StataCorp LLC).

## Results

**Figure 1.**
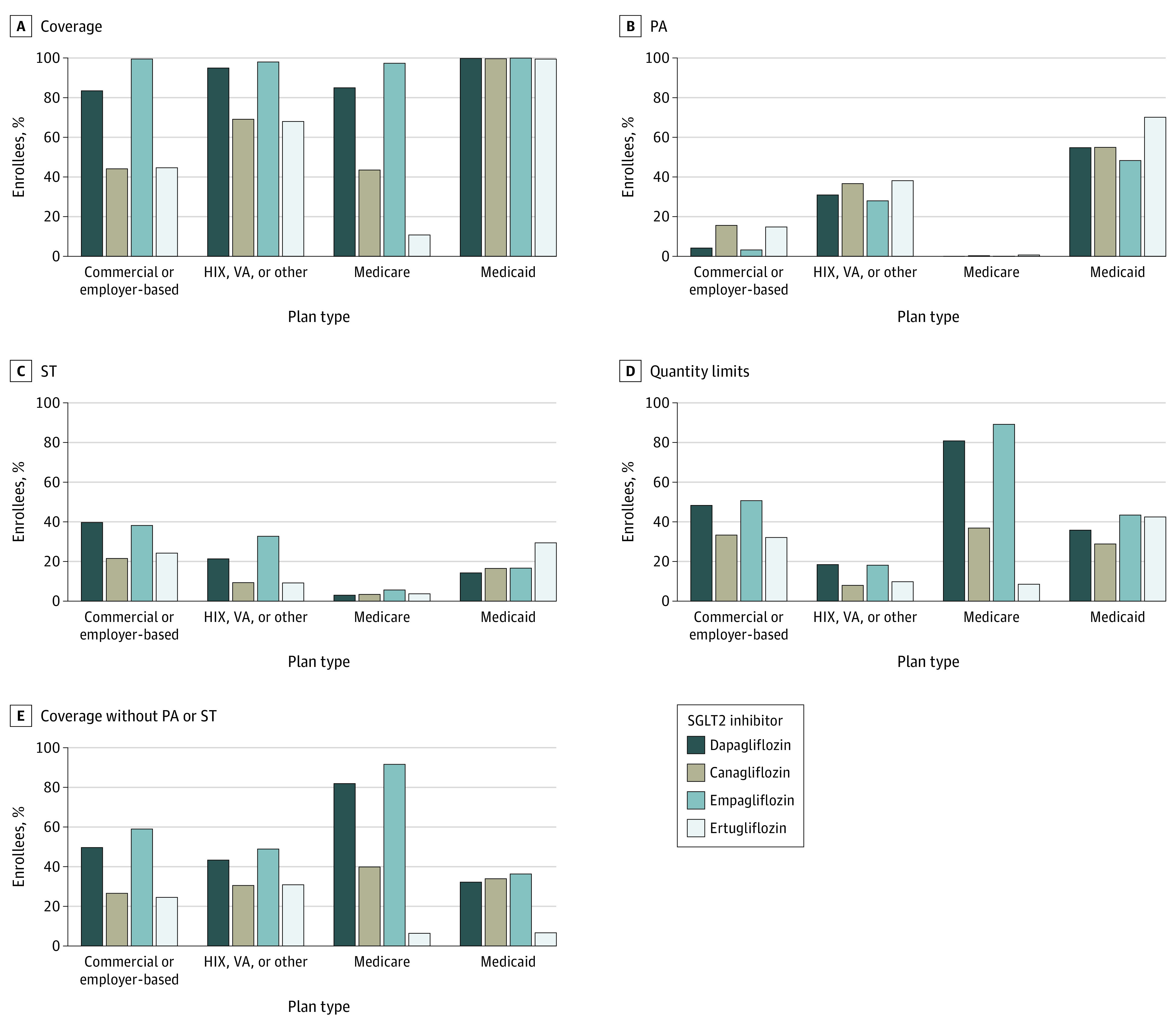
Percentage of Health Plan Enrollees With Sodium-Glucose Cotransporter 2 (SGLT2) Inhibitors Coverage and Formulary Restrictions by Plan Type Data were obtained from Fingertip Formulary (Clarivate Analytics LLC). Coverage, prior authorizations, step therapy, and quantity limits differed across plan types for all SGLT2 inhibitors (*P* < .001 for all comparisons). Health insurance exchange (HIX), Veterans Affairs (VA), or other plans (other plans include Federal Employees Health Benefits program, non-VA federal program, municipal plan, pharmacy benefit manager, and union plans). Medicare plan type includes Medicare Advantage, Medicare Part D, Medicare Special Needs, and Employer Group Waiver Plans. Medicaid plan type includes managed Medicaid, Medicare-Medicaid, Programs of All-Inclusive Care for the Elderly, and state Medicaid. Dual-eligible patients typically receive prescription drug coverage through Medicare Part D plans. PA indicates prior authorization; ST, step therapy.

We examined plan-level data from a total of 4135 US health plans, including 1842 commercial or employer-based plans; 892 HIX, VA, or other plans; 860 Medicare plans; and 541 Medicaid plans, accounting for 297 850 145 covered lives. Coverage varied across SGLT2 inhibitors and plan types (Figure 1). Medicaid plans were most likely and Medicare plans were least likely to require prior authorization. For example, prior authorization was required for empagliflozin for 48% of Medicaid covered lives, compared with 0.1% of Medicare covered lives. Step therapy requirements were most frequent in commercial or employer-based plans (eg, 40% of covered lives for dapagliflozin) and least frequent in Medicare plans (3% of covered lives for dapagliflozin). Medicare plans were most likely to cover at least 1 SGLT2 inhibitor without requiring prior authorization or step therapy (98% of covered lives), followed by commercial or employer-based plans (61% of covered lives); HIX, VA, or other plans (51% of covered lives); and Medicaid plans (43% of covered lives).

**Figure 2.**
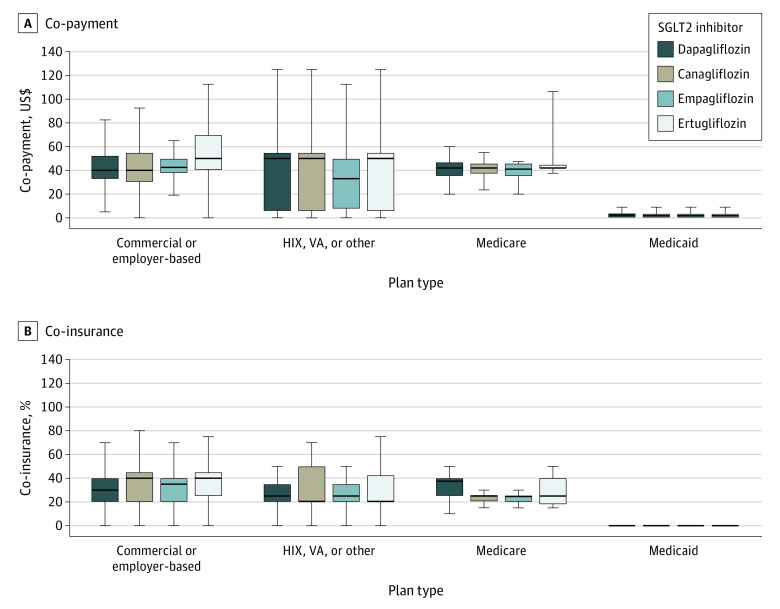
Cost Sharing for Sodium-Glucose Cotransporter 2 (SGLT2) Inhibitors by Plan Type Data were obtained from Fingertip Formulary (Clarivate Analytics LLC). Box plots contain the median, 25th and 75th percentiles, and lower and upper adjacent values. Co-payments for all SGLT2 inhibitors were higher under commercial or employer-based; health insurance exchange (HIX), Veterans Affairs (VA), or other plans and Medicare plans compared with Medicaid plans (*P* < .001 in unadjusted linear regression). Expected co-payment information was missing in 2143 plans (52%) for dapagliflozin, 3018 plans (73%) for canagliflozin, 1876 plans (45%) for empagliflozin, and 3273 plans (79%) for ertugliflozin. Expected co-insurance information was missing in 3243 plans (78%) for dapagliflozin, 3647 plans (88%) for canagliflozin, 3201 plans (77%) for empagliflozin, and 3712 plans (90%) for ertugliflozin. Expected co-insurance information was missing in Medicaid plans.

Among plans with data on expected co-payments and co-insurance (423 [10%] to 2259 [55%] of 4135 plans), median monthly co-payments and co-insurance were similar across commercial or employer-based; HIX, VA, or other; and Medicare plans (Figure 2). Compared with other plan types, co-payments were much lower in Medicaid plans (median [IQR] cost, $2.28 [$0-$3.90] for dapagliflozin; *P* < .001).

## Discussion

Across 4135 US health plans, we found substantial formulary restrictions, high co-payments, and high co-insurance to control the use of SGLT2 inhibitors. Commercial and employer-based plans most often used step therapy requirements, whereas HIX and Medicaid plans often required prior authorization. Medicare plans were the most likely to cover at least 1 SGLT2 inhibitor without prior authorization or step therapy, whereas Medicaid plans were the least likely to do so. Individuals with Medicaid faced the greatest formulary restrictions and the lowest out-of-pocket costs for SGLT2 inhibitors. Study limitations included a high rate of missing data for co-payment and co-insurance to characterize the out-of-pocket costs for SGLT2 inhibitors.

These results demonstrate that considerable barriers to access and affordability of SGLT2 inhibitors exist for patients with chronic kidney disease, heart failure, and diabetes. Such barriers may contribute to delays in care, greater administrative waste, lower medication adherence,^5^ disparities in SGLT2 inhibitor uptake,^1^ and worse health outcomes.^5^

Because the benefits of SGLT2 inhibitors accrue over a long time horizon, payers may not have the incentive to bear their costs, despite the cost-effectiveness and potential cost savings associated with these drugs.^6^ Efforts to align incentives across the payer, pharmaceutical, patient, and health care system perspectives may speed the adoption of this crucial therapeutic class and promote health care value.
